# Incidence and Characteristics of Nonfatal Opioid Overdose Among Youths Aged 11 to 24 Years by Sex

**DOI:** 10.1001/jamanetworkopen.2020.30201

**Published:** 2020-12-17

**Authors:** Sarah M. Bagley, Mam Jarra Gai, Joel J. Earlywine, Samantha F. Schoenberger, Scott E. Hadland, Joshua A. Barocas

**Affiliations:** 1 Division of General Pediatrics, Department of Pediatrics, Boston University School of Medicine, Boston, Massachusetts; 2 Grayken Center for Addiction, Department of Medicine, Boston Medical Center, Boston, Massachusetts; 3 Section of General Internal Medicine, Department of Medicine, Boston University School of Medicine, Boston, Massachusetts; 4 Section of Infectious Diseases, Boston Medical Center, Boston, Massachusetts; 5 Department of Health Law, Policy, and Management, Boston University School of Public Health, Boston, Massachusetts

## Abstract

**Importance:**

Opioid-related overdose has substantially increased among adolescents and young adults in recent years. How overdose differs by age and sex among youths and the factors associated with overdose by sex remain poorly described.

**Objective:**

To compare the sociodemographic and clinical characteristics of female and male youths who have experienced a nonfatal opioid overdose (NFOD) and compare the incidence of NFOD by sex.

**Design, Setting, and Participants:**

This retrospective cohort study used data on US individuals aged 11 to 24 years with a diagnosis of NFOD from the IBM MarketScan Commercial Database from January 1, 2006, through December 31, 2017.

**Exposure:**

Sex.

**Main Outcomes and Measures:**

The primary outcome was NFOD stratified by sex; covariates included sociodemographic and clinical characteristics.

**Results:**

Among 20 312 youths aged 11 to 24 years who had a history of NFOD and met study eligibility criteria, the median age was 20 years (interquartile range, 18-22 years; mean [SD] age, 20.0 [2.9] years) and 56.7% were male. Compared with male youths, female youths had a higher baseline prevalence of mood or anxiety disorder (65.5% vs 51.9%, *P* < .001), trauma and stress-related disorders (16.4% vs 10.1%, *P* < .001), and history of suicide attempt or self-harm (14.6% vs 9.9%, *P* < .001). Male youths had a higher prevalence of opioid use disorder (44.7% vs 29.2%, *P* < .001), cannabis use disorder (18.3% vs 11.3%, *P* < .001), and alcohol use disorder (20.3% vs 14.4%, *P* < .001). The incidence rate ratio of NFODs in females vs males was greater than 1 for ages 11 to 16 years and was less than or equal to 1 after age 17 years.

**Conclusions and Relevance:**

This cohort study found differences between female and male youths in sociodemographic and clinical characteristics and incidence of NFOD. Although female and male youths who experience overdose appear to have different risk factors, many of these risk factors may be amenable to early detection through screening and intervention.

## Key Points

**Question:**

Among youths who experience nonfatal opioid overdose, are there differences by sex in sociodemographic and clinical characteristics and incidence of occurrence?

**Findings:**

In this cohort study of 20 312 commercially insured US youths aged 11 to 24 years who experienced nonfatal opioid overdose, female youths had a higher prevalence of baseline anxiety, depression, and history of self-harm; male youths had a higher baseline prevalence of other substance use disorders. Among those aged 11 to 16 years, female youths experienced a significantly greater incidence of nonfatal opioid overdose than male youths; after age 17 years, the incidence became greater for male youths than for female youths.

**Meaning:**

This study found differences between female and male youths in sociodemographic and clinical characteristics and incidence of nonfatal opioid overdose, suggesting a need for tailored strategies to address overdose in this population.

## Teaser

This cohort study compares the sociodemographic and clinical characteristics of US youths who have experienced a nonfatal opioid overdose and examines the incidence of nonfatal opioid overdose by sex.

## Introduction

The prevalence of opioid use disorder (OUD) and number of fatal opioid-related overdoses have significantly increased among adolescents and young adults in recent years. The rate of diagnosis of OUD increased 6-fold from 2001 to 2014 among youths.^1^ However, only 1 in 4 youths with OUD receives timely (within 3-6 months) treatment after diagnosis.^1,2^ In parallel with the increasing prevalence of OUD, the percentage of heroin-related overdoses among adolescents aged 15 to 19 years increased 404% from 1999 to 2016.^3^ The number of opioid-related overdose deaths was 4 times higher in 2018 than in 1999 among individuals aged 18 to 25 years, and 12% of all deaths in this age group were from opioid-related overdose.^3,4,5^ Despite evidence of the significant consequences that the opioid crisis has had on youths, the factors associated with overdose among youths, including age and sex, are not established. Many questions about the specific characteristics of youths who experience an overdose remain unanswered.

Among adults, a greater proportion of opioid overdoses occur in men^5,6^; however, opioid overdose deaths have significantly increased among women over the past 20 years. The percentage of prescription opioid deaths increased 583% among women compared with 404% among men from 1999 to 2016. Among women aged 20 to 64 years, the percentage of opioid overdose deaths increased 260% from 1999 to 2017, and there was an increase of 1643% in deaths involving synthetic opioids.^7^ The causes of these increases may be different for women than for men.^8^ For example, women report using opioids to cope with pain and negative emotions more commonly than men.^9,10^ Co-occurring psychiatric disorders are more prevalent among women than men,^11^ with women more likely to report experiencing a traumatic event and the onset of posttraumatic stress disorder before the development of a substance use disorder.^12,13,14^ Furthermore, women entering treatment for substance use typically present with a profile of more severe medical, behavioral, psychological, and social problems than men despite a shorter duration of use.^11,15^

However, few studies have examined whether such sex-based differences in opioid overdose risk extend to the population of adolescents and young adults. In a study of 478 street-involved youths (those with no fixed address for 3 consecutive nights or who had not been staying with parents or caregivers in the previous 6 months) aged 14 to 26 years, being female was associated with a higher risk of overdose.^16^ Another retrospective analysis of 58 treatment-seeking youths aged 16 to 26 years revealed a higher risk of previous overdose among female youths compared with male youths.^17^ Neither of these studies specifically examined differences potentially associated with the higher risk among female youths. Although more men than women aged 18 years or older have alcohol and other substance use disorders, girls and boys younger than 18 years have approximately the same prevalence of use.^18^ At earlier ages, no sex differences in the prevalence of current alcohol use and binge drinking are apparent.^18^ In addition, adolescent girls report higher rates of past-month use of prescription opioid than boys.^18^ Girls may be at elevated risk for depression, anxiety, and trauma, which are established risk factors for substance use.^19,20^ Furthermore, girls might initiate substance use for different reasons than boys. Reports of drug use to cope with mood disturbances and elevated anxiety are more strongly associated with substance use behaviors in women than in men.^15^ Boys report sensation seeking as a main motivation for using drugs. Girls report using drugs to increase confidence, reduce tension, cope with stress, decrease inhibitions, and manage weight.^9,10,21^

Considering that opioid overdose risk is different among adult women and men and that small-sample data suggest a higher risk of overdose among girls than among boys, sex-based differences among individuals who experience opioid overdose should be characterized. These data are needed to develop and implement effective interventions that are responsive to specific risk profiles among all youths. The objectives of this study were to compare (1) the characteristics of female and male youths who have experienced a nonfatal opioid overdose (NFOD) and (2) the incidence of NFOD between female and male youths.

## Methods

### Study Design and Cohort

We conducted a retrospective cohort study using the IBM MarketScan Commercial Database, which includes all inpatient, outpatient, emergency department, behavioral health, and prescription drug claims from more than 150 million unique individuals with employer-provided health insurance in the US between January 1, 2006, and December 31, 2017. Eligible individuals were aged 11 to 24 years and had experienced an NFOD during the study period. We defined opioid overdose using *International Classification of Diseases, Ninth Revision* (*ICD-9*) and *International Statistical Classification of Diseases and Related Health Problems, Tenth Revision (ICD-10)* diagnosis codes.^22^ The study was not considered human subjects research by the Boston University School of Medicine institutional review board. This study followed the Strengthening the Reporting of Observational Studies in Epidemiology (STROBE) reporting guideline.

### Variables

Study covariates included patient factors selected a priori based on their previously identified or hypothesized associations with NFOD.^16,17,23^ Patient factors included age at the time of overdose, sex, urban or rural residence based on living inside or outside a metropolitan statistical area, mood or anxiety disorder, psychosis, attention-deficit/hyperactivity disorder, trauma or stress-related condition, conduct disorder or antisocial personality disorder, other substance use disorder (tobacco, stimulant, alcohol, or cannabis), history of suicide attempt or self-harm, and acute and chronic pain condition based on *ICD-9* and *ICD-10* diagnosis codes.^22,24^ These covariates were identified using diagnosis codes during a 12-month observation period on or before the NFOD. In addition, we included new diagnosis of OUD in the 90 days after an NFOD, new claim for medication for opioid use disorder (MOUD), and recurrent NFOD. MOUD was defined as sublingual buprenorphine, oral naltrexone, and injectable naltrexone in the 90 days after an NFOD. Until late 2017, methadone therapy was not covered by commercial insurance; therefore, it is not reliably captured in this data set and was not included in our analysis.

### Statistical Analysis

We used descriptive statistics to characterize the sample with regard to patient factors. All analyses were stratified by sex. We used the χ^2^ test to compare factors among individuals with NFOD, the median number of recurrent NFODs, and the proportion of youths who received a new diagnosis of OUD or were prescribed MOUD after an NFOD. Next, we calculated the proportion of youths with NFODs by age group (11-12 years, 13-14 years, 15-16 years, 17-18 years, 19-20 years, 21-22 years, and 23-24 years). We calculated the incidence rate and incidence rate ratio by age. Time at risk was restricted to enrollment period and individuals aged 24 years or younger. Analyses were conducted using SAS, version 9.4 (SAS Institute Inc). Statistical tests were 2-tailed and considered significant at *P* < .05.

## Results

### Overall Cohort Characteristics

Among 20 312 youths aged 11 to 24 years who had a history of NFOD and met study eligibility criteria, the median age was 20 years (interquartile range, 18-22 years; mean [SD] age, 20.0 [2.9] years) and 56.7% were male (Table 1). Psychiatric diagnoses were common among youths: 57.8% had mood and anxiety disorders, 12.8% had trauma- or stress-related disorders, and 11.7% had attention-deficit/hyperactivity disorder. At the time of NFOD, 38% of youths had OUD, 28% had nicotine use disorder, 17.7% had alcohol use disorder, and 15.2% had cannabis use disorder.

**Table 1.** Baseline Characteristics of Commercially Insured US Youths With a First Nonfatal Opioid Overdose From 2006 to 2017

Characteristic	Youths, No. (%) (N = 20 312)
Sex	
Male	11 527 (56.7)
Female	8785 (43.3)
Age, y	
11-12	133 (0.7)
13-14	691 (3.4)
15-16	2109 (10.4)
17-18	3249 (16.0)
19-20	4538 (22.3)
21-22	5009 (24.7)
23-24	4583 (22.6)
Urban metropolitan statistical area	17 840 (87.8)
Mood or anxiety disorder	11 738 (57.8)
Psychosis	1363 (6.7)
Trauma- and stress-related disorders	2596 (12.8)
Nicotine use disorder	5691 (28.0)
Cannabis use disorder	3094 (15.2)
Alcohol use disorder	3602 (17.7)
Attention-deficit/hyperactivity disorder	2380 (11.7)
Conduct disorder and antisocial personality disorder	870 (4.3)
Opioid use disorder	7712 (38.0)
Stimulant use disorder	1951 (9.6)
History of suicide attempt or self-harm	2421 (11.9)
Chronic pain	11 472 (43.5)
Acute pain	5139 (25.3)

### Cohort Characteristics by Sex

Among individuals who had experienced an NFOD, being female was associated with having a mood or anxiety disorder (65.5 vs 51.9%, *P* < .001), trauma- and stress-related disorder (16.4% vs 10.1%, *P* < .001), history of suicide attempt or self-harm (14.6% vs 9.9%, *P* < .001), and chronic pain (62.1% vs 52.2%, *P* < .001) (Table 2). Being male was associated with having attention-deficit/hyperactivity disorder (13.2% vs 9.8%, *P* < .001), OUD (44.7% vs 29.2%, *P* < .001), nicotine use disorder (31.0% vs 24.2%, *P* < .001), cannabis use disorder (18.3% vs 11.3%, *P* < .001), stimulant use disorder (10.8% vs 8.1%, *P* < .001), and alcohol use disorder (20.3% vs 14.4%, *P* < .001). Prevalence of mood or anxiety disorder among male youths was 51.9%, and 10.1% had a trauma- or stress-related disorder. Most youths (84.5%) did not have a recurrent NFOD, although female youths were less likely than male youths to have a recurrence of NFOD.

**Table 2.** Baseline Characteristics of Youths With First Nonfatal Opioid Overdose and Receipt of Medication Within 6 Months, Stratified by Sex^a^

Characteristic	Youths, No. (%)		*P* value
	Female (n = 8785)	Male (n = 11 527)	
Urban metropolitan statistical area	7619 (86.7)	10 221 (88.7)	<.001
Mood or anxiety disorder	5758 (65.5)	5980 (51.9)	<.001
Psychosis	615 (7.0)	748 (6.5)	.15
Attention-deficit/hyperactivity disorder	861 (9.8)	1519 (13.2)	<.001
Trauma and stress-related disorders	1438 (16.4)	1158 (10.1)	<.001
Conduct disorder and antisocial personality disorder	362 (4.1)	508 (4.4)	.32
Use disorder			
Opioid	2562 (29.2)	5150 (44.7)	<.001
Nicotine	2127 (24.2)	3564 (31.0)	<.001
Cannabis	990 (11.3)	2104 (18.3)	<.001
Stimulant	709 (8.1)	1242 (10.8)	<.001
Alcohol	1263 (14.4)	2339 (20.3)	<.001
History of suicide attempt or self-harm	1285 (14.6)	1136 (9.9)	<.001
Chronic pain	5453 (62.1)	6019 (52.2)	<.001
Acute pain	2054 (23.3)	3084 (26.8)	<.001
Receipt of medication^b^			
Any medication	519 (5.9)	1080 (9.4)	.61
Buprenorphine	378 (4.3)	809 (7.1)	.37
Oral naltrexone	147 (1.7)	277 (2.4)	.26
Injectable naltrexone	30 (0.3)	78 (0.7)	.28

^a^ Reported characteristics are from 12 months before diagnosis of nonfatal opioid overdose.

^b^ Participants did not receive medication treatment in the 30 days before nonfatal opioid overdose. Receipt of more than 1 medication in the 6 months after overdose was possible.

Of the 20 312 in the cohort, 7.5% (1516 of 20 312 youths) received a new diagnosis of OUD after an NFOD and 32% (486 of 1512) were female. Most diagnoses were made within 30 days of an NFOD (402 of 486 female [82.7%]; 864 of 1030 male [83.9%]).

Among 1599 youth who received MOUD within 90 days of an NFOD, 519 (32%) were female. Buprenorphine was the most common MOUD prescribed for both female and male youths (378 of 519 female [73%]; 809 of 1080 male [75%]; *P* < .61) (Table 2).

### Proportion and Incidence of NFOD by Sex and Age

**Figure 1.**
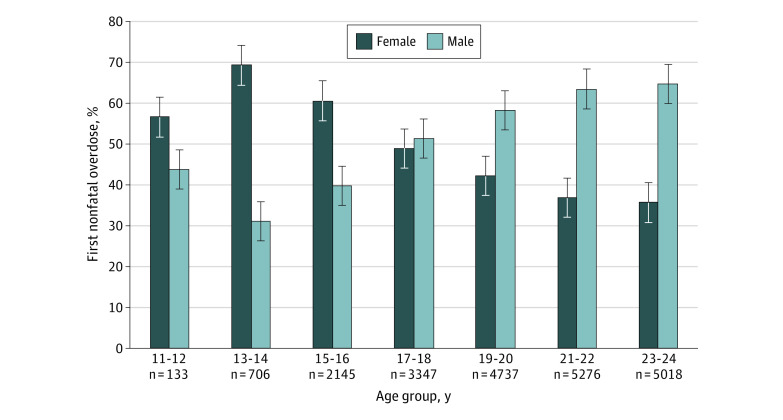
Distribution of First Nonfatal Opioid Overdose by Age and Sex

**Figure 2.**
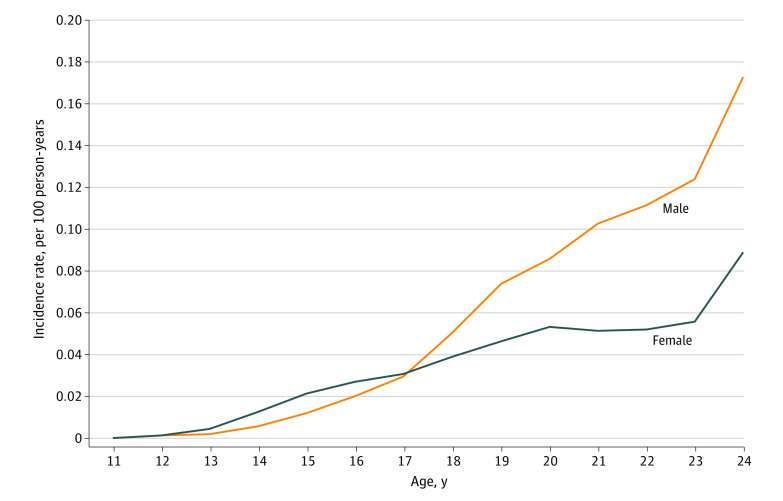
Incidence Rate of First Nonfatal Opioid Overdose by Age and Sex from 2006 to 2016

**Figure 3.**
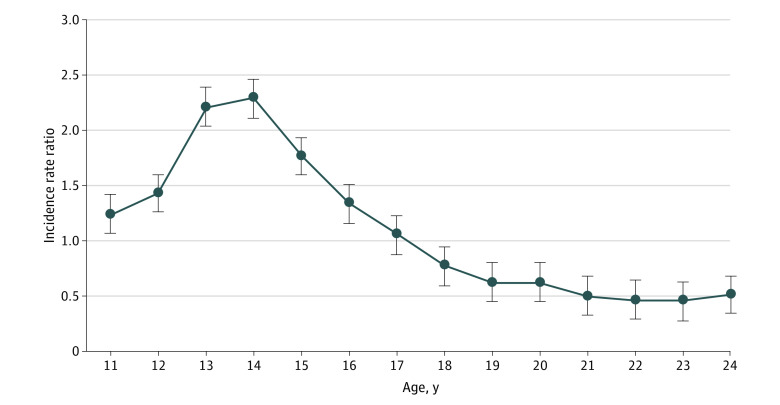
Incidence Rate Ratio of First Nonfatal Opioid Overdose by Sex from 2006 to 2016 The ratio is female to male.

Of 3042 NFODs among youths aged 11 to 17 years, 1825 (60%) occurred in females (Figure 1). Starting at age 17 years, a greater proportion of NFODs occurred in males. The incidence rate of NFOD was significantly higher among female youths aged 11 to 16 years (0.009 vs 0.005 per 100 person-years), but for youths 17 to 24 years of age, the incidence rate became higher among male youths (0.048 vs 0.083 per 100 person-years) (Figure 2). The incidence rate ratio of female to male NFOD was greater than 1 for ages 11 to 16 years and was 1 or less after age 17 years (Figure 3).

## Discussion

In this cohort study of commercially insured US youths, we found differences between male and female youths in the factors associated with NFOD and the ages at which these overdoses occurred. Significant differences were found with regard to comorbid psychiatric illness and substance use disorders between males and females. In addition, the incidence rate of NFOD was significantly higher among female youths until 17 years of age and among male youths older than 17 years. Fewer than 1 in 10 youths received MOUD in the 6 months after an NFOD, and only 7.5% received a new diagnosis of OUD.

We found that female and male youths had a high prevalence of co-occurring but different psychiatric illness and substance use disorders. Although in our study, female youths had a lower prevalence of all substance use disorders, including OUD, and a higher prevalence of mood and trauma-associated disorders, both male and female youths had a higher prevalence of psychiatric illness and substance use disorder than youths in the general population. Our findings are consistent with previous studies^15,19,20^ that demonstrated that females (both girls and women) had higher rates of depression and anxiety. These other studies also showed that females reported drug use to reduce negative affect^10,25,26^ and as a maladaptive coping mechanism for trauma.^27,28^ Further research is needed to expand on the association that we identified between psychiatric illness and first NFOD and to explore how youths’ psychiatric disorders affect their risk for NFOD. Such research could have a substantial effect on intervention development for this population. Furthermore, although psychiatric diagnoses were more prevalent among females, 52% of males had a mood or anxiety disorder and 10% had a trauma- or stress-related disorder. Understanding the potential sex-based differences will be critical to appropriate targeting of interventions. Our findings may have implications for follow-up care. Nonfatal overdoses did not occur in isolation from other psychiatric and substance use disorders. Much of the research on reducing overdose mortality is focused on expansion of the immediate treatment for overdose (naloxone) and MOUD, which have been shown to be associated with reduced mortality in adults.^29,30,31^ Although these interventions are critical, further research to test strategies to integrate treatment for co-occurring psychiatric and other substance use disorders for youths is needed. Furthermore, screening for these other disorders using adolescent-validated tools, including screening for history of trauma and self-harm and offering early interventions, may be valuable for identifying potential risk for NFOD.

Our finding that females younger than 17 years had a higher incidence of NFOD is consistent with epidemiologic data that have indicated changes in alcohol and drug prevalence among female youths.^32^ For many years, alcohol and drug use was more common among male youths. However, in recent years, a narrowing of the gender gap in use of certain substances has been noted. According to 2019 Monitoring the Future data, female youths in the eighth and tenth grade had a higher prevalence of cannabis, amphetamine, and tranquilizer use.^32^ Deeper understanding of the trajectory of subsequent substance use and treatment for female youths as well as sex-specific approaches to prevention are needed. If, for instance, trauma is associated with NFOD in this population, programmatic change could identify persons with a history of trauma and help prevent trauma from occurring. In addition, if co-occurring mental illness is associated with NFOD, reexamination of how to improve access to and utilization of mental health services among youths may be needed. After the age of 17 years, although the incidence rate increased among both females and males, it was higher among males, and the difference between the rates was larger.

Having a diagnosis of conduct or antisocial personality disorder was the only characteristic that was not significantly different between females and males. However, some of the differences in factors, such as acute pain and urban residence, were small and may not be clinically relevant. When interventions to address NFOD in youths are being designed, consideration of the size of these differences and the clinical implication will be important.

Youths in this cohort had a low rate of medication treatment, which is consistent with prior studies highlighting the important and often missed opportunity to engage them in care after an opioid overdose.^33,34^ However, less than one-third of females (29%) had an identified diagnosis of OUD at the time of the overdose, and only 32% of patients who received new OUD diagnoses were female. It is not possible to know from this sample whether these findings were attributable to underdiagnosis of OUD or whether NFOD was associated with high-risk opioid use not meeting the threshold of OUD. Nevertheless, these results highlight potential sex-based differences that warrant further investigation to understand disparities.

### Limitations

This study has limitations. First, the cohort consisted only of commercially insured youths and should be replicated with publicly insured and uninsured youths. Although other studies of youths have demonstrated that the prevalence of OUD among youths with private insurance is similar to that among youth with public health insurance, prevalence and risk for NFOD may differ by insurance status.^1,2^ In a study of Medicaid-enrolled youths who experienced NFOD, a higher prevalence of co-occurring mental health disorders was observed, highlighting the need to offer integrated care for substance use and mental health disorders.^33^ Second, this cohort included only youths who sought health care after an NFOD. It is possible that youths who do not seek care have different characteristics that may lead to different conclusions about necessary interventions. Third, substance use and mental health disorders frequently go undiagnosed in youths, and the actual prevalence may be different from what we found. Furthermore, it is possible that diagnoses of mental health disorders were given without clinical assessments and may have been overdiagnosed. However, prior studies have demonstrated a high prevalence of co-occurring substance use and mental health disorders. Fourth, race data in this database are unreliable, and thus we did not include race as a variable. It is imperative to ensure that databases and future studies provide data about race/ethnicity. Fifth, we were unable to characterize gender identity in this database and were limited to sex as listed by an insurance carrier. We recognize that there are likely gender-related factors associated with NFOD, particularly among persons who identify as transgender or gender nonconforming, that we were unable to assess using these data. Databases should also provide data about gender identity.

## Conclusions

In this cohort study of youths with NFOD, we found significant sex- and age-based differences in the prevalence of types of co-occurring mental health and substance use disorders and the incidence of NFOD. These differences may have important implications for developing effective interventions to prevent first-time NFOD and to engage youths in care after an NFOD. Sex- and age-based risk should be considered in strategies to improve opioid overdose prevention and medication treatment access for youths.
